# Can a single pulse transcranial magnetic stimulation targeted to the motor cortex interrupt pain processing?

**DOI:** 10.1371/journal.pone.0195739

**Published:** 2018-04-09

**Authors:** Lee-Bareket Kisler, Ilan Gurion, Yelena Granovsky, Alon Sinai, Elliot Sprecher, Simone Shamay-Tsoory, Irit Weissman-Fogel

**Affiliations:** 1 Department of Psychology, University of Haifa, Haifa, Israel; 2 Laboratory of Clinical Neurophysiology, Technion Faculty of Medicine, Haifa, Israel; 3 Department of Neurology, Rambam Health Care Campus, Haifa, Israel; 4 Department of Neurosurgery, Rambam Health Care Campus, Haifa, Israel; 5 Faculty of Social Welfare and Health Sciences, University of Haifa, Haifa, Israel; University Medical Center Goettingen, GERMANY

## Abstract

The modulatory role of the primary motor cortex (M1), reflected by an inhibitory effect of M1-stimulation on clinical pain, motivated us to deepen our understanding of M1’s role in pain modulation. We used Transcranial Magnetic Stimulation (TMS)-induced virtual lesion (VL) to interrupt with M1 activity during noxious heat pain. We hypothesized that TMS-VL will effect experimental pain ratings. Three VL protocols were applied consisting of single-pulse TMS to transiently interfere with right M1 activity: (1) VL_M1_- TMS applied to 11 subjects, 20 msec before the individual’s first pain-related M1 peak activation, as determined by source analysis (sLORETA), (2) VL_-50_ (N = 16; TMS applied 50 ms prior to noxious stimulus onset), and (3) VL_+150_ (N = 16; TMS applied 150 ms after noxious stimulus onset). Each protocol included 3 conditions ('pain-alone', ' TMS-VL', and ‘SHAM-VL’), each consisted of 30 noxious heat stimuli. Pain ratings were compared, in each protocol, for TMS-VL vs. SHAM-VL and vs. pain-alone conditions. Repeated measures analysis of variance, corrected for multiple comparisons revealed no significant differences in the pain ratings between the different conditions within each protocol. Therefore, our results from this exploratory study suggest that a single pulse TMS-induced VL that is targeted to M1 failed to interrupt experimental pain processing in the specific three stimulation timing examined here.

## Introduction

Studies show that the primary motor cortex (M1) can modulate pain by influencing its affective or sensory components or by top down activation of the periaqueductal gray (PAG) [1–6]. Determining M1’s inhibitory role in pain processing motivated clinicians and researchers to use M1 stimulation for analgesic purposes [7–12]. Intracortical recordings [13] and functional neuro-imaging studies have reported pain-related changes in M1 activity [14, 15], in addition to activation of complex network of cerebral structures, associated with different dimensions of pain [16, 17]. However, if M1 is activated during pain process it does not mean that interruption of its activity will necessarily disrupt pain processing.

An experimental approach to study the functional contribution of a cortical area in a given task is 'Virtual lesion’ (VL), induced by applying brief transcranial magnetic stimulation (TMS) during a task performance [18, 19]. VL can be achieved either by using a single or double TMS pulse [19–24] or by a short train of repetitive TMS (rTMS) [25–28] time locked to a stimulus or a task. The magnetic pulse operates as 'neural noise', adding random activity that interrupts the neural activity of the cortical region [29–32]. The effect of single pulse VL lasts between 50–200 msec following stimulus onset, reducing the cortical neural activity [19, 27]. Thus, VL causes transient and reversible interference with cortical processing and is therefore used to examine a region’s involvement in a particular task [18, 33]. In order to achieve maximal effect, it is best to apply the VL to the desired cortical target during its engagement in the task [34–38].

We therefore aimed in this study to apply TMS-induced VL in order to deepen our understanding of the role of M1 in pain modulation. We used 3 VL-TMS protocols in two separated data collections. Each protocol was time-locked to the stimulus onset or M1 activation (determined from pain evoked potential recordings using M1 as a region-of-interest [ROI] analysis). We hypothesized that TMS-VL to M1 will disrupt pain processing resulting in changes in pain ratings to experimental noxious stimuli as compared to baseline (pain-alone) and VL-sham conditions.

## Material and methods

### Subjects

Following the exclusion of 3 subjects due to high levels of resting motor threshold (see following), 11 subjects (all females, age: 24.5±2.4 years) participated in VL_M1_ protocol and 16 subjects (8 females and 8 males, age: 25.3±1.6 years) participated in the VL_-50_ and VL_+150_ protocols (these two protocols were performed in one session) (see ‘TMS protocols and conditions’ for protocols description). All were right handed. Participants were asked to avoid drinking alcohol and taking analgesics 24 hours before the experiment, as well as to refrain from drinking caffeine 3 hours prior to the experiment. Exclusion criteria were: any significant acute pain during the last 3 months; any chronic pain condition and/or; any metabolic, psychiatric or neurological disorders. Particular emphasis was put on epilepsy, convulsive seizures during childhood or first degree relative suffering from epilepsy. Since stimulus intensity was set to 120% of the individual’s resting motor threshold (rMT), to reduce the risk of seizure, participants with rMT greater than 54% of the TMS machine output were not recruited. This criterion excluded 3 subjects. The experimental protocol was approved by the institutional review board (IRB) of the University of Haifa (approval # 191/13). All participants signed an informed consent prior to beginning the experiment. Participants were recruited by advertisement adds, posted at the University of Haifa. Data collection was performed at the University of Haifa, Haifa, Israel in two separate experiments, from Sep 2013 till Feb 2014 for the VL_M1_ protocol, and from Nov 2014 till Jan 2015 for the VL_-50_ and VL_+150_ protocols.

### Pain psychophysics

Instrument: For the application of the heat stimuli, we used the PATHWAY sensory evaluation system (Medoc, Ramat-Yishai, Israel) with the 572.5 mm^2^ thermode. The thermode heating and cooling rate is 70°C/sec and 40°C/sec, respectively.

Familiarization: In order to familiarize subjects with the experimental procedure, and to train them using pain ratings, four series of 3 brief heat stimuli were applied to the left, non-dominant volar forearm. The three-series consisted of a baseline temperature of 42°C and a destination temperature of 45, 48 and 52°C, in a pseudo-randomized order. Subjects rated the pain intensity on a numerical pain scale (NPS) of 0–100 (0-indicated "no pain" and 100-related to the "maximum imaginable pain "). All subjects rated the 52°C noxious stimulus higher than 30 and the innocuous baseline temperature (constant 42°C) lower than 15, on the NPS.

The contact heat stimuli: In each TMS protocol, three series of brief contact heat stimuli were administered to the left non-dominant volar forearm. Each series consisted of 30 brief heat stimuli with an inter-stimulus interval ranging from 8–12 sec [39–41]. The baseline and peak temperatures were 42 and 52°C, respectively. We used a baseline temperature of 42°C because it was shown to improve the detection of contact heat evoked potentials (CHEPs) [42, 43] (recorded in the pain-alone condition in theVL_M1_ protocol, see below_)_ that is attributed to an increased synchronization of afferent volley and reduced inter-trial variability [42, 43]. In order to minimize habituation and sensitization the thermode was slightly moved to an adjacent area from one stimulus to the other [44]. Three seconds following the stimulus onset, a sound prompted the subject to rate his/her pain on a 0–100 NPS.

### TMS and EMG instrument

TMS: TMS stimuli were applied using the Magventure system (MagProX100, Magventure Tonika Elektronic, Farum, Denemark) with a passive cooling figure-of-eight coil (MCF-B65).

EMG: Electromyography (EMG) were recorded by an EMG amplifier module (Magventure Tonika Elektronic) from the left abductor pollicis brevis (APB) using surface electrodes.

### TMS protocols and conditions

**Fig 1:**
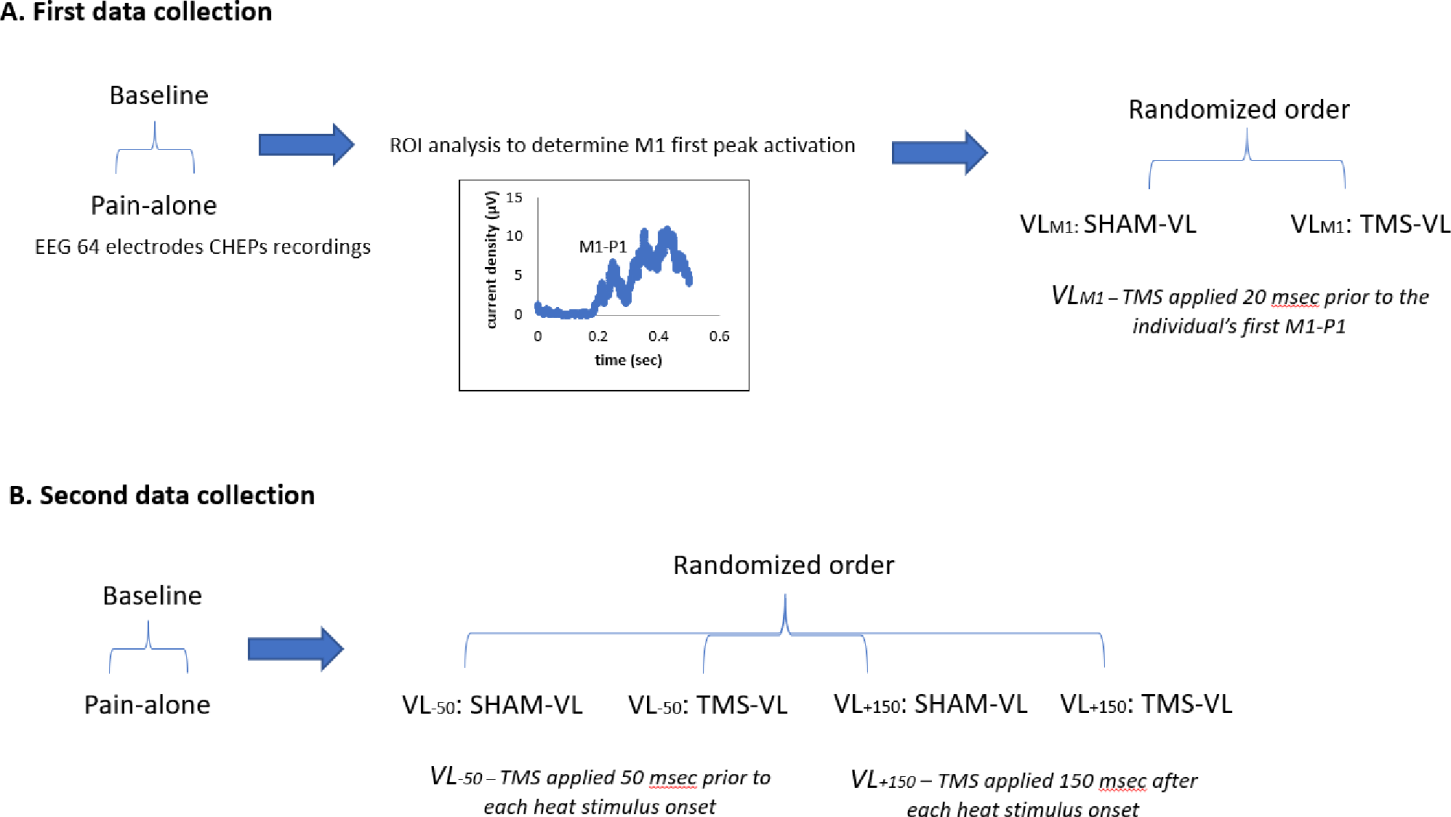
The 3 VL protocols. Illustration of the conditions and order in the (A) VLM1 data collection (first data collection) and (B) VL-50, VL+150 data collection (second data collection). Pain-alone–no TMS; TMS-VL–active TMS; SHAM-VL–the TMS coil turned upside down.

**Fig 2:**
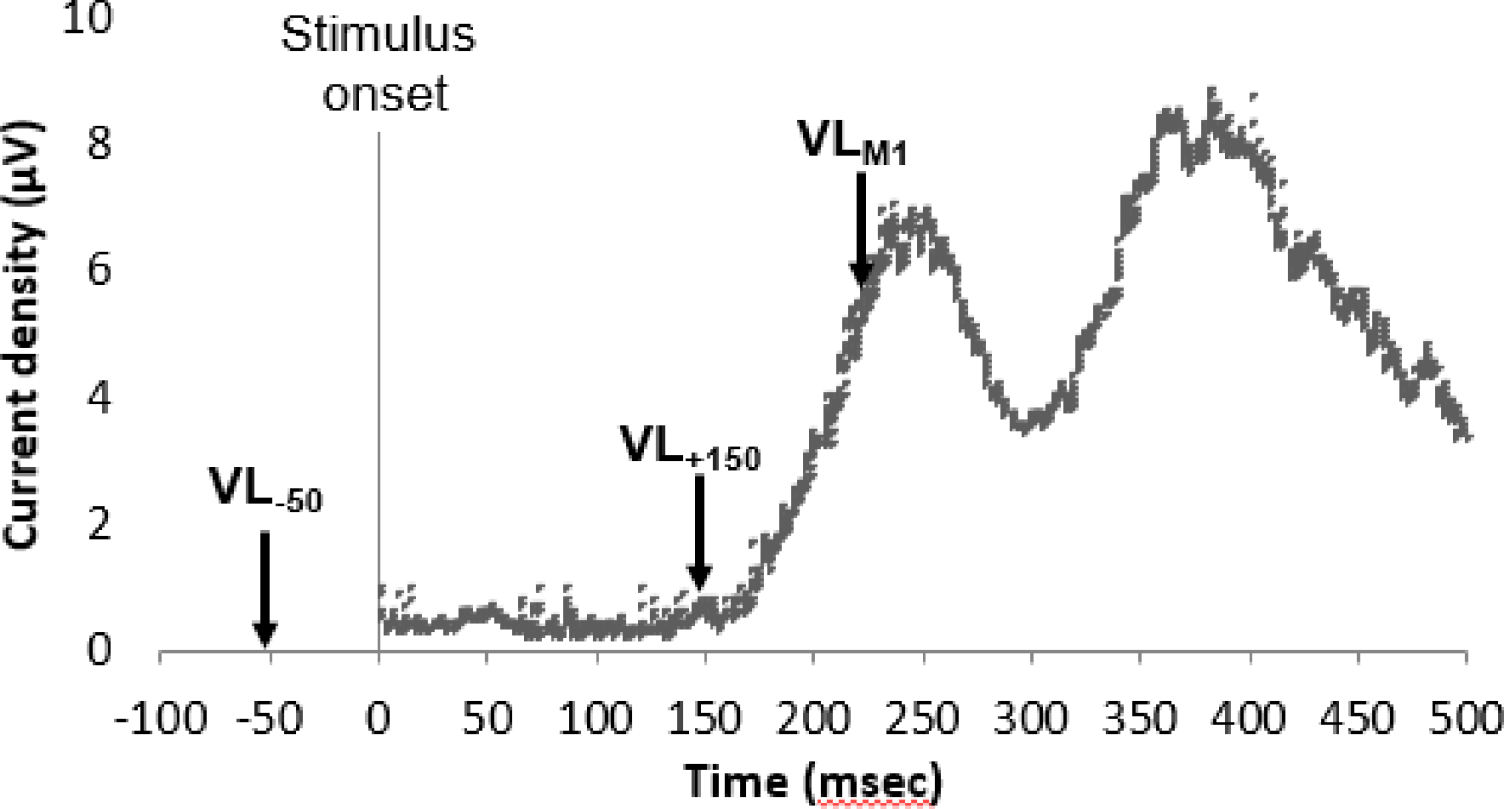
The 3 VL protocols stimulation timing. Illustration of the stimulation timing in the 3 different protocols relative to the current density in the primary motor cortex. In the VL_M1_, the TMS was applied 20 msec preceding the first peak of M1’s pain related activity. In VL_-50_ and VL_+150_, the TMS pulse was applied 50 msec prior and 150 msec after the noxious heat stimulus onset, respectively.

The study included three TMS protocols (VL_M1_, VL_-50_ and VL_+150_) (see Figs 1 and 2). In each protocol the TMS stimuli were applied at intensity of 120% of the rMT, administered to the individually selected spot at the right hemispheres' M1-maximal APB response. The rMT was determined using the EMG response. During stimulation, the position of the coil and EMG response was monitored by an assistant to make sure the coil remained in the same spot. Each protocol included three conditions (Fig 1): 1) 'pain-alone'- pain stimuli were delivered with no TMS; 2) 'TMS-VL'–active TMS and 3) 'SHAM-VL'–performed with the TMS coil turned upside down, 180° [45]. The flipped coil lies tangent to the subjects' M1 stimulation area, while the front part of the coil, where the stimulation is maximal turns to the other direction, away from the subjects' head. The coil used in this study, is 6.3cm thick with the active coil at one side. Therefore, when the coil is turned upside down (sham condition) the active coil becomes much distant compared to the condition of active TMS. The resulted stimulation intensity is reduced, by roughly 60%, according to the manufacturer. We successfully used this sham stimulation method in a previous VL study (see Granovsky et al. 2016 [45]). The pain-alone condition was performed at the beginning of the session and used as baseline. Thereafter, the TMS-VL and SHAM-VL were performed in a randomized order.

*VL*_*M1*_ protocol–First, CHEPs recordings were performed during the pain-alone condition. Based on previous sLORETA (standardized low-resolution brain electromagnetic tomography) [46, 47] region of interest (ROI) analysis from our lab that focused specifically on M1 activity during similar stimuli (Kisler et al. 2017; see more details below), the first M1 peak activation was determined. The TMS stimuli were then applied, in the TMS-VL and SHAM-VL conditions, 20 msec prior to the individual’s first M1 peak activation, induced by each heat stimulus. *VL-50*—The TMS stimuli were applied 50 msec prior to each heat stimulus onset. *VL+150*—The TMS stimuli were applied 150 msec after each heat stimulus onset.

### CHEPs recording and processing

Instrument: Electroencephalography (EEG)-64 channels system was used for the CHEPs recording (*EASYCAP*—*Fast*'*nEasy Cap*- Brain products GmbH, Munich, Germany) in the VL_M1_ protocol. Stimulus onset was marked by a square TTL (transistor-transistor logic) wave of 100 msec, sent from the PATHWAY system to the EEG system at the beginning of the temperature rise. The TTL was marked along with the EEG recording in separate channel concomitant to the temperature rise.

EEG recording: During CHEPs recordings, subjects were asked to stay still, relax their muscles and keep their eyes open. CHEPs recording was performed with a bandpass of 0.1–1000 Hz and a sampling rate of 5000 Hz. Impedances of all electrodes were kept below 5 kΩ.

EEG processing (in VL_M1_ protocol, performed following the pain-alone condition): The recorded EEG data during the pain-alone condition, were analyzed using BrainVision analyzer version 2.0.2 (Brain products GmbH, Munich, Germany). Electrodes were visually inspected and noisy ones were disabled and removed from further analysis. The average recorded neural activity from all remaining channels was used as a reference. Recorded data was filtered with a bandpass of 0.1–70 Hz, divided into epochs of 1500 msec. Each epoch included 400 msec prior to the stimulus onset for baseline correction and 1100 msec post stimulus onset. Epochs contaminated with eye blinks or other artifacts were rejected from averaging through a semi-automatic artifact rejection procedure. The remaining epochs (mean ±SD: 24.73±2.97) were averaged.

Identification of the individual M1 peak activity: The individual M1 neural activity associated with the heat stimuli, in the VL_M1_ protocol, was estimated using ROI analysis in sLORETA following the pain-alone condition. The ROI, consisting of two right hemispheres' M1 voxels (x = 30, y = -20, z = 45; x = 35, y = -20, z = 45; voxel size: 5mm^3^) was chosen based on a previous study with similar CHEPs recording, specifically looking at M1 [48]. The average current density of 0.2 msec time frames between 0–500 msec after stimulus onset was extracted from the ROIs and analyzed in Excel. Based on our previous study [48], that showed M1 first peak activity (maximum amplitude) occurs during 230–300 msec following stimulus onset, the timing of the TMS pulse was determined relative to the individual's M1 first peak activity, within this time frame. In order to achieve maximal interference with the M1 ongoing activity, the TMS pulse was applied 20 msec prior to the individual M1 first peak activity [35].

### Experimental procedure

The experiment included 2 independent data collections (Fig 1), with different subjects; one data collection consisted of one session for the VL_M1_ protocol including CHEPs recording during the pain-alone condition and a second data collection consisted of one session that included both the VL_-50_ and VL_+150_ protocols in a randomized order_._ Each session comprised of pain-alone performed at baseline, followed by TMS-VL, and SHAM-VL conditions of the tested TMS protocol. Ten minutes break was kept between conditions. The participants were blinded to the experimental conditions and order. At the beginning of each session, participants underwent a familiarization with the experimental tests. Thereafter, the rMT was evaluated, defined as the lowest intensity to evoke a response >50 μV in 5 out of 10 trials. rMT estimation was performed using motor evoked potentials (MEP) recorded from the APB muscle. This spot was used for the TMS induced VL applied at intensity of 120% rMT.

### Statistical analyses

Statistical analysis was performed using SPSS 19 and JMP 12.1.0 Based on a previous study that used a similar protocol [49], a power analysis was performed, which indicated that, in order to achieve similar effects, with correction for multiple comparisons and power of 0.80, a total of 8 subjects would be required. This analysis yielded 8 participants to observe an effect, if existed. Importantly, we did not aim to examine differences between the different protocols but only differences between the three conditions within the protocols. In order to examine VL effect on pain ratings, the averaged pain ratings in each condition (active TMS (TMS-VL); sham TMS (SHAM-VL); pain-alone) was compared using a repeated measure analysis of variance (ANOVA). This was done for each of the TMS protocols. If needed, post hoc analysis was performed with Bonferroni correction. The results of the analysis are presented as mean ± standard deviation. A p value below 0.05 was considered significant in all statistical tests.

## Results

### Cortical analysis: Determining TMS stimulation timing in the VL_M1_ protocol

Based on ROI analysis, M1 showed two pain related peak activation (see Fig 2) [48]. The latency of M1’s first peak amplitude (M1-P1) was identified for each individual. The mean M1-P1 amplitude and latency were 10.92±12.38 and 251.82±16.45 msec, respectively, and thus the timing for the TMS stimulation (i.e. 20 msec before the individual M1-P1 peak activation) was 231.82±16.45 msec on average (see Fig 2).

### TMS stimulation characteristics and effect on psychophysics

**Fig 3:**
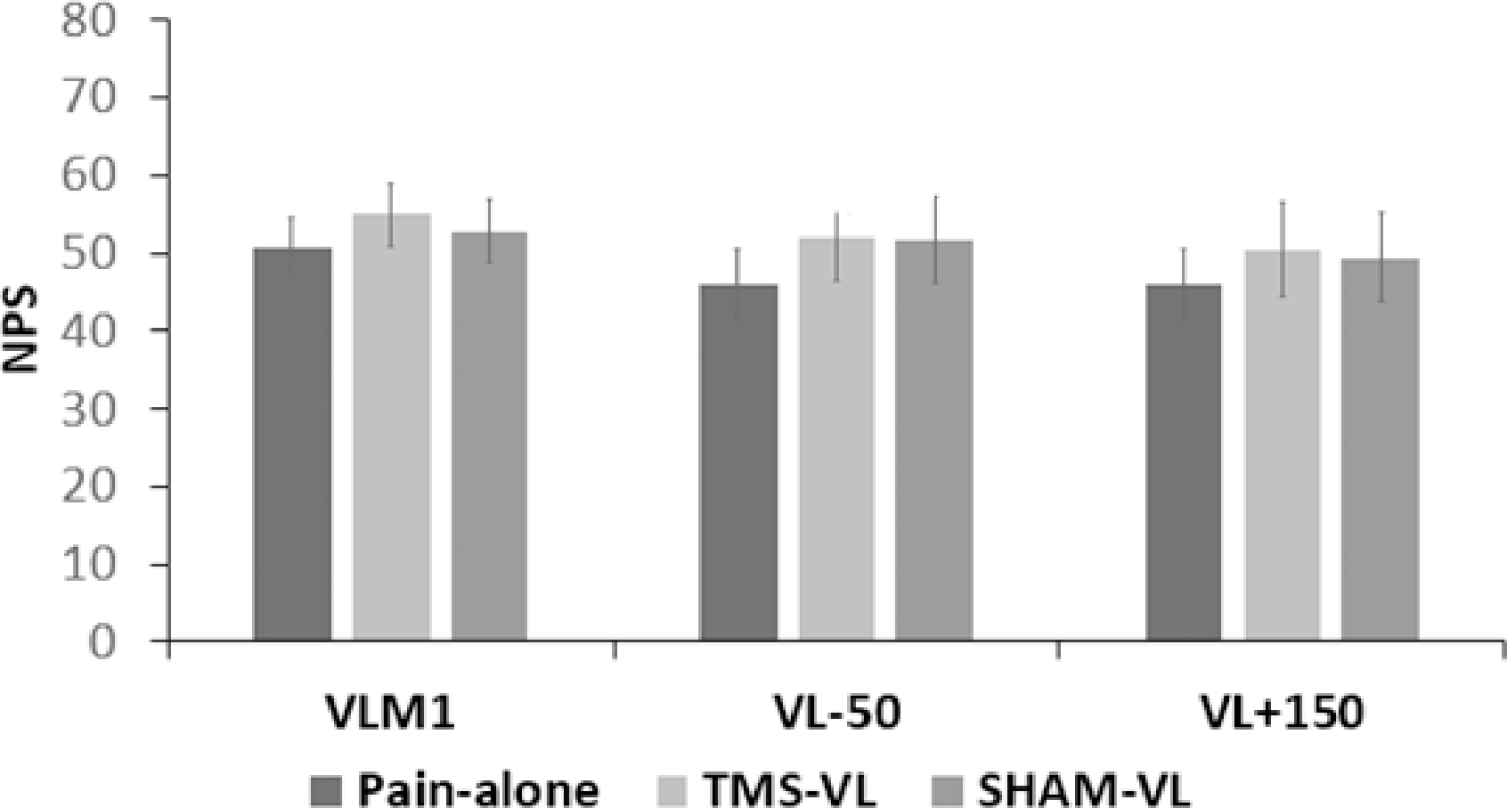
Pain ratings in the TMS protocols. After correcting for multiple comparisons, no significant differences were found between the different conditions in the 3 protocols. Data are presented as mean ± SE.

In the VL_M1_ protocol, the average rMT was 46.5±6.7% from the maximal TMS output, and the average stimulation intensity was therefore 55.9±8.1%. In the VL_-50_ and VL_+150_ protocols, the average rMT was 50.3±3.9% from the maximal TMS output, and the average stimulation intensity was therefore 60.4±4.7%. Repeated measures ANOVA revealed no differences between the three different conditions for the VL_M1_ and VL_+150_ protocols (VL_M1_: F(2,20) = 0.95; P = 0.405; VL_+150_: F(2,30) = 1.56; P = 0.227). However, a significant effect was revealed for the VL_-50_ protocol (F(2,30) = 4.52; P = 0.019). Post hoc analysis showed a significant difference between the pain-alone and SHAM-VL conditions (P = 0.021). However, following Bonferroni correction for multiple comparisons, this was no longer significant (P = 0.063) (Table 1 and Fig 3).

**Table 1:** Pain ratings (mean ± SD) in the TMS protocols.

		Condition (Mean ± SD)			Repeated measures ANOVA P-value
		pain-alone	TMS-VL	SHAM-VL	
Protocol	VL_M1_	50.8±12.9	54.9±13.0	52.8±13.4	0.405
	VL_-50_	46±17.6	51.9±21.8	51.8±22	0.019*
	VL_+150_		50.5±23.9	49.5±22.8	0.227

VL- virtual lesion; TMS- transcranial magnetic stimulation; VL_M1_- TMS applied 20 msec prior to the first peak of M1’s pain related activity; VL_-50_ and VL+150—TMS applied 50 msec prior and 150 msec after noxious heat stimulus onset, respectively.

*P<0.05

## Discussion

In this study, we aimed to deepen our understanding of the role of M1 in pain modulation using TMS-induced VL. We tried three different stimulation timing to interrupt M1 activity at different stages: (1) 50 msec prior to the noxious heat stimulation onset in order to interfere with M1 incoming activity, (2) 150 msec following the stimulus onset aiming at interfering with the M1 first peak activation (centered at 250 msec- see Fig 2), and (3) 20 msec before the individual M1 first peak activation for the purpose of impeding the ongoing M1 pain-related activity. We found that VL to M1 as applied in these three protocols, in our study has no effect on experimental pain intensity ratings.

Research using VL assumes that if a cortical brain area is essential for a specific function than a lesion, in our case a VL, will disturb this function, resulting in dysfunction [19, 33]. Importantly, the advantage of a VL induced by a single pulse TMS is that its effect is time-specific, and it can therefore reveal the involvement of the targeted region in different task related processing stages [33]. In this study, after correcting for multiple comparisons, all three VL protocols did not show significant differences between the different conditions. Previous findings indicate that TMS to M1 induces inhibitory postsynaptic potential for 50–200 msec, impeding the cortical activity [19, 27]. Accordingly, we hypothesized that early TMS stimulation, 50 msec before the onset of noxious heat stimulation, can interfere with the incoming M1 activity and thus impair early stages of pain processing buildup. The peak of this early pain processing is reflected in the early pain evoked potential (N1) that occurs at about 200 msec following contact heat pain stimulation [43] and is associated with sensory-discriminative processes that take place at SI and SII [50–52]. A study that used several time intervals for stimulation was conducted by Kanda et al (2003). They applied pairs of TMS pulses to several areas, among them the sensorimotor cortex (not M1), at several time intervals. They found increased pain sensitivity, when the TMS pulses were applied 150–200 msec following laser stimulation. Therefore, we also assumed that applying TMS time-locked to the individual M1 activation (20 msec before M1’s first peak or 150 msec following stimulation onset), may influence late stages of pain processing, at 250–400 msec after stimulus onset (i.e. N2-P2 [43]; subjective pain experience [39, 51]). However, this effect was not confirmed. The difference in our findings, compared to those of Kanda et al (2003), could result from longer and possibly augmented effect on neuronal activity when applying paired pulse TMS, as compared to a single pulse [19, 53, 54].

Lin et al. (2012) applied single pulse TMS to M1 at 50 or 100 msec prior to an electrical stimulation and found an effect on pain ratings lasting up to 15 minutes after stimulation [49]. However, the study design suffers from a few limitations, which make it hard to interpret the results. For example, the real TMS condition always preceded sham TMS condition and no randomization was applied. Since the protocol of this study was similar to ours, we used its' data, despite of its drawbacks, for power analysis. This resulted in the number of 8 participants per protocol. Since the number of participants in each of the three protocols exceeded 8 participants (11 for the VLM1 and 16 for the VL-50 and VL+150 protocols) we believe our results reflect true negative findings yet limited to the specific protocol and timing examined here, during brief noxious heat stimuli. Therefore, it is possible that single pulse TMS stimulation at other timing (e.g. +50, +100) would yield different results and further studies are needed.

In the current study, the VL effect was not significantly different than sham-VL. It is possible that the sham-VL condition, which included upside-down placing of the coil, does not completely eliminate the TMS effect [55, 56] and a different sham, such as stimulating a brain area that is not involved in pain processing [23, 57], is required. Nonetheless, similar to Granovsky et al., [45] that employed a similar sham protocol but for rTMS, subjects were naïve to the aim of the study and blinded to the stimulation condition, supported by similar cortical sensations and stimulation sounds. Another methodological consideration is that the TMS stimuli were applied to the APB cortical representation, while the noxious stimuli were given to the forearm. Considering the overlap in the stimulated M1 cortical area that results in a motor response both in the APB and forearm muscle (e.g. flexor carpi radiali—FCR) [58–60], the overlap in M1's somatotopic organization of the arm and hand muscles [61], and that TMS stimulation affects nearby areas [27, 33], we expected that stimulating the APB will also affect the forearm [60]. Moreover, Wassermann et al., (1992) showed similar rMT in the APB and FCR. Therefore, relying on the hand rMT as a reference to stimulation intensity is expected to provide a good estimate for the forearm rMT. Of note, our measure for a VL effect relied entirely on the subject's pain rating. The lack of a VL effect on pain ratings does not rule out a VL effect on neural activity, which was not directly examined here. In fact, as previous studies show, a VL effect on neural activity is not always evident behaviorally [34]. Nonetheless, as pain ratings is the gold standard in evaluating pain, we chose this as our main measure and based on Lin et al., (2012) that used a similar VL protocol, we expected our protocol to affect the pain ratings. Importantly, this kind of VL protocol is well established and widely used to induce VL effect in many areas other than pain [23, 27, 33, 57, 62–64]. Specifically, a single suprathreshold TMS stimulus to M1 first generates a short synchronization of neural activity, followed by inhibition [27, 32]. This is one of the indications of a VL effect. Furthermore, functional imaging of suprathreshold TMS stimulation to M1 showed a distributed network of brain activity including the M1, thalamus, insula, cingulate and somatosensory (SI and SII) cortices [65, 66]. Therefore, though no effect was found on pain ratings in our study, it is suggested that our protocols influenced the neural activity in M1 and in remote pain-related brain areas. Another possibility to the lack of observed effect on pain ratings is the use of brief noxious contact heat stimuli. However, as these stimuli were shown to be modulated in response to an extra segmental conditioning stimulus [67], we believe this is not the case. Another factor to consider is the possibility that sex may interfere with the VL effect. Females were shown to display greater VL response in a previous study that used rTMS [45]. Our cohort for the VL_M1_ protocol consisted of only females and we did not find an effect on pain sensitivity. The other two VL protocols included both males and females. Thus, it is speculated that the lack of effect in the other VL protocols is a result of a mixed group of females and males. However, considering our small cohort number, we do not think that testing both sexes impaired our ability to observe a VL effect.

We are the first to apply VL time-locked to M1 activity (VLM1) in relation to pain processing, however, similar intervention was already applied to other brain areas that have a role in different functions [21, 37, 38, 68–70]. While some studies used time-locked single pulse TMS [20], other used pairs of TMS pulses [21, 22] or a train of stimuli (rTMS) [28, 71] in order to interfere with the cortical area’s function. This raises a question regarding the optimal TMS paradigm that is required for a VL. Furthermore, contrary to other sensory processes that take place in one primary sensory cortex (i.e. tactile, auditory, visual), pain is a more complex experience, processed in a network of cortical areas (i.e. primary (SI) and secondary (SII) somatosensory cortex, M1, anterior cingulate cortex, insula, and prefrontal cortices such as dorso-lateral, medial, and orbital). It is therefore suggested that since M1 is not the 'primary pain area', a single-pulse VL is not robust enough with the timing used, to have an effect on pain ratings and a more powerful VL intervention or different timing is required, one that will cause a significant disruption of the pain system function [45].

Importantly, though not identical to VL, a recently published sham-controlled, double-blind cross-over study using HF-rTMS to M1 in healthy participants found no difference in pain intensity compared to sham [28]. In another supportive study, Borckardt et al. (2011) employed different high and low frequency rTMS protocols to M1, on various experimental noxious stimuli including warm and cold sensory and pain threshold, as well as suprathreshold stimuli. The main analgesic effects were observed on sensory and pain thresholds with no significant effect on suprathreshold thermal pain ratings. The authors concluded that the effect of rTMS on pain intensity, in healthy subjects is “small and variable”. However, an effect was seen on suprathreshold unpleasantness ratings [25]. It is therefore possible that the modulatory role of M1 is related to the affective-motivational aspect of pain [72] which was not tested in our study, though this is also controversial [28, 73].

To summarize, this study examined M1’s involvement in pain perception using single pulse TMS. In order to enlighten M1’s role in pain processing, we used 3 different single pulse VL-TMS protocols targeted to M1 and found no effect on pain ratings. As the pain experience is complex and consists of a large network of brain areas connected to M1, it is suggested that with the timing used here, a single pulse TMS is not powerful enough to induce an observable effect on pain ratings and a more intense intervention or different stimulation timing is required.

## Supporting information

S1 File Data file -three protocols. (XLSX)
